# Development of Loop-Mediated Isothermal Amplification Assay Targeting *lytA* and *psaA* Genes for Rapid and Visual Diagnosis of *Streptococcus pneumoniae* Pneumonia in Children

**DOI:** 10.3389/fmicb.2021.816997

**Published:** 2022-01-17

**Authors:** Shuheng Du, Chao Yan, Bing Du, Hanqing Zhao, Guanhua Xue, Ping Zheng, Yanling Feng, Jinghua Cui, Lin Gan, Junxia Feng, Zheng Fan, Ziying Xu, Tongtong Fu, Hanyu Fu, Qun Zhang, Nannan Li, Rui Zhang, Shiyu Liu, Xiaoran Li, Xiaohu Cui, Yao Zhou, Qi Zhang, Yaodong Chen, Jing Yuan

**Affiliations:** ^1^Department of Bacteriology, Capital Institute of Pediatrics, Beijing, China; ^2^Key Laboratory of Resources Biology and Biotechnology in Western China, Ministry of Education, College of Life Sciences, Northwest University, Xi’an, China; ^3^College of Biomedicine, Beijing City College, Beijing, China

**Keywords:** *Streptococcus pneumoniae*, loop-mediated isothermal amplification, detection, visual, children

## Abstract

*Streptococcus pneumoniae* (*S. pneumoniae*) is a common major human pathogen associated with community-acquired pneumonia, septicemia, meningitis, and otitis media. It is difficult to isolate and identify *S. pneumoniae* form clinical samples. To evaluate a novel, rapid, sensitive, and specific loop-mediated isothermal amplification (LAMP) assay to detect *S. pneumoniae* pneumonia in children, we designed specific LAMP primers targeting *lytA* and *psaA* genes. We optimized the reaction time and reaction system, and evaluated its sensitivity and specificity of detection using real-time turbidity monitoring and visual observation. We also analyzed the molecular characteristics of the isolates obtained from the positive samples. The primer sets LytA-1 and PsaA-2 amplified the genes in the shortest times, and 63°C was confirmed as the optimum reaction temperature. The detection sensitivity of each reaction was 10 and 100 copies/μL with primer sets LytA-1 and PsaA-2, respectively. This LAMP assay showed no cross-reactivity with other 27 pathogens. To describe the availability of this method, we collected 748 clinical samples from children with pneumonia. Among them, 135 were confirmed to be *S. pneumoniae* positive by LAMP. The sensitivity was 100% (95% CI 96.4–100%), specificity 99.0% (95% CI 97.8–99.6%). Including them, 50 were co-infected with *Mycoplasma pneumoniae*. This LAMP assay detected *S. pneumoniae* in 1 h and the results can be identified with visual naked eyes. Thus, it will be a powerful tool for *S. pneumoniae* early diagnosis and effective antibiotic therapy.

## Introduction

*Streptococcus pneumoniae* (*S. pneumoniae*) is a Gram-positive encapsulate diplococcus and conditional pathogen (Sadowy and Hryniewicz, 2020). It usually spreads to the upper respiratory tract in aerosol form through droplets and colonizes the mucosal surface of the host nasopharynx and upper airway, without major clinical symptoms (Paton and Trappetti, 2019). The carriage rate of *S. pneumoniae* in children aged 2–3 years is the highest, and in school-age children, it accounts for 20–60% pneumonia (Walker et al., 2013). It is one of the main pathogens of community-acquired pneumonia, and is a leading cause of various pneumococcal diseases, such as acute otitis media, sepsis, and meningitis (Brown and Lerner, 1998). It remains the leading infectious cause of childhood morbidity and mortality worldwide in emergency or refugee settings, especially in developing countries (O’Brien et al., 2009). The incidence and global burden of severe pneumonia are highest in southeast Asia and Africa. Hence, it is crucial to identify *S. pneumoniae* accurately for early diagnosis and better treatment.

The organism produces multifaceted virulence factors, including the capsule, cell wall, choline-binding proteins, cell-surface proteins, pneumolysin (ply), autolysin (lytA), and the metal-binding protein pneumococcal surface antigen A (psaA). *S. pneumoniae* can spread from the nasopharynx and upper airway to the lower respiratory tract and cause pneumonia, through the combined activity of several virulence factors (Weiser et al., 2018).

Conventional diagnosis of *S. pneumoniae* infection mainly relies on culture and molecular-based testing (Fierz, 2004; Nilsson et al., 2008; Muñoz-Almagro et al., 2011; Kosack et al., 2017; Arguedas et al., 2020). Moreover, it is difficult to distinguish *S. pneumoniae* from other closely related *Streptococcus* spp. with similar biochemical properties (Tabatabaei et al., 2021). To popularize the monitoring of pathogens, it is necessary to develop a simple and low-cost method for pathogen detection.

In recent years, isothermal amplification detection has been widely used in the detection of various pathogenic microorganisms (Wong et al., 2018). Loop-mediated isothermal amplification (LAMP) originally developed by Notomi et al. is a novel, highly sensitive, and specific method for diagnosis of infectious diseases (Notomi et al., 2000). It has become a research hotspot in the field of nucleic acid detection and diagnosis because of its speed, simplicity, and no need for precise instruments (Chen et al., 2011; Patel et al., 2014). LAMP has been widely used in the detection of influenza virus, severe acute respiratory syndrome coronavirus 2, Middle East respiratory syndrome coronavirus, West Nile virus, Ebola virus, Zika virus, yellow fever virus, and many other pathogens (Ge et al., 2013; Cao et al., 2016; Huang et al., 2018; Yan et al., 2020). LAMP technology overcomes the dependence of traditional PCR temperature cycling on thermostable enzymes and reaction equipment, and has good sensitivity. Additionally, the results can be observed with the naked eye (Notomi et al., 2000).

In this study, an efficient and accurate LAMP method was developed for the detection of *S. pneumoniae* and validated *via* real-time PCR and PCR-sequencing. We designed five pairs of primers for *lytA* and *psaA* genes. We established a rapid, visual and efficient LAMP method for the detection of *S. pneumoniae* by optimizing the reaction system and conditions. Finally, we analyzed 748 clinical samples collected from children for clinical diagnosis of pneumonia.

## Materials and Methods

### Viruses, Bacterial Strains, and Clinical Samples

The new LAMP assay was evaluated with 20 clinically common pathogens: influenza A and B viruses, parainfluenza viruses, adenoviruses, respiratory syncytial virus, human metapneumovirus, human bocavirus, rhinovirus, *Mycoplasma pneumoniae* (*M. pneumoniae*) M129, *M. pneumoniae* FH, *Haemophilus influenzae*, *Legionella pneumophila*, *Listeria monocytogenes*, *Staphylococcus aureus*, *Klebsiella pneumoniae*, *Pseudomonas aeruginosa*, *Escherichia coli, Acinetobacter baumannii*, *Mycobacterium tuberculosis*, and *Bordetella pertussis*. *Streptococcus mitis*, *Streptococcus oralis*, *Streptococcus agalactiae*, *Streptococcus mutans*, *Streptococcus parasanguinis*, *Streptococcus sanguinis*, and *Streptococcus salivarius* were also used to evaluate the new LAMP assay. Viral and bacterial types used in this study listed in Table 1.

**TABLE 1:** Viral and bacterial types used in this study.

Species	Source
Influenza A	Our microorganism center
Influenza B	Our microorganism center
Parainfluenza viruses (PIV)	Our microorganism center
Adenoviruses (ADV)	Our microorganism center
Respiratory syncytial virus (RSV)	Our microorganism center
Human metapneumovirus (HMPV)	Our microorganism center
Human bocavirus (BoV)	Our microorganism center
Rhinovirus (Rh)	Our microorganism center
*Mycoplasma pneumoniae* M129	Our microorganism center
*Mycoplasma pneumoniae* FH	Our microorganism center
*Streptococcus pneumoniae* ATCC49619	Our microorganism center
*Haemophilus influenzae* ATCC10211	Our microorganism center
*Legionella pneumophila* ATCC33823	Our microorganism center
*Listeria monocytogenes*	Clinical isolate
*Staphylococcus aureus* ATCC29213	Our microorganism center
*Klebsiella pneumoniae* ATCC BAA-2146	Our microorganism center
*Pseudomonas aeruginosa*	Clinical isolate
*Escherichia coli* ATCC25922	Our microorganism center
Acinetobacter baumannii	Clinical isolate
*Mycobacterium tuberculosis*	Clinical isolate
*Bordetella pertussis*	Clinical isolate
*Streptococcus mitis*	Clinical isolate
*Streptococcus oralis*	Clinical isolate
*Streptococcus agalactiae*	Clinical isolate
*Streptococcus mutans*	Clinical isolate
*Streptococcus parasanguinis*	Clinical isolate
*Streptococcus sanguinis*	Clinical isolate
*Streptococcus salivarius*	Clinical isolate

A total of 748 clinical specimens were collected from pediatric patients at the Affiliated Children’s Hospital of the Capital Institute of Pediatrics in Beijing, China, from January 2019 to September 2021. Each specimen was obtained from a separate patient. The specimens included bronchoalveolar lavage fluid, throat swabs, and sputum samples.

### DNA Extraction

Total DNA was extracted from the specimens using the QIAamp DNA Mini Kit (Qiagen, Hilden, Germany). The extracted DNA was stored at -80°C.

### Preparation of Artificial *lytA* and *psaA* Genes

Virulence genes *lytA* and *psaA* were further screened out as the two target genes using diverse data sources at NCBI^1^. The whole sequence of *lytA* (957 bp) and *psaA* (930 bp) corresponding to the nucleotide sequences of *S. pneumonia* R6 (GenBank accession number: AE007317) were cloned into the pEASY-T1 vector (Tiangen Biochemical Technology Co., Ltd.). The plasmid concentration was measured with a Thermo Scientific NanoDrop spectrophotometer. The copy number of the recombinant plasmid was calculated. Tenfold serial dilutions of the recombinant plasmid ranging from 10^8^ to 1 copy/μL were prepared.

### Primer Design

The sequences of *lytA* and *psaA* genes in different serotypes were further screened (Supplementary Figure 1). Five sets of LAMP primer sequences targeting *lytA* and *psaA* were designed separately using online software PrimerExplorer V5.^2^ A set of five/six primers was designed, mainly for seven/eight different regions of the target gene: external forward primer (F3), external reverse primer (B3), forward internal primer (FIP), reverse internal primer (BIP), loop forward primer (LF), and loop backward primer (LB). The primers were synthesized by Sangon Biotech (Shanghai) Co. Ltd. and purified by high-performance liquid chromatography.

### Loop-Mediated Isothermal Amplification Reaction

The LAMP reaction was performed using the Loopamp DNA amplification kit (Eiken Chemical Co., Ltd., Tokyo, Japan). The reaction volume of 25 μL contained 12.5 μL 2 × Reaction Mix, 1.0 μL Bst DNA polymerase 1, 40 pmol primers FIP and BIP, 5 pmol primers F3 and B3, 20 pmol primers LF and/or LB, 1 μL fluorescent detection reagent (when needed), and 2 μL template DNA. The mixture was incubated at an isothermal temperature 63°C for 60 min. The process was monitored using a Loopamp Real-time Turbidimeter (LA-230; Eiken Chemical Co., Ltd., Tochigi, Japan). Turbidity readings at optical density 650 nm were obtained every 6 s, and the reaction was considered positive when the turbidity values were >0.1. For visual observation, 1 μL fluorescent calcein was added to the mixture, and a color change from orange to green was observed by the naked eye for a positive reaction.

### Real-Time PCR and PCR-Sequencing

The published real-time PCR assay based on the *lytA* gene were performed as described previously (Carvalho Mda et al., 2007). The oligonucleotide sequences of the *lytA*-CDC forward primer (5′-ACGCAATCTAGCAGATGAAGCA-3′), *lytA*-CDC reverse primer (5′-TCGTGCGTTTTAATTCCAGCT-3′), and *lytA*-CDC probe (5′-FAM-GCCGAAAACGCTTGATACAGGGAG-3′-BH Q1) were used. This assay were carried out in a reaction volume of 25 μL by use of the TaqMan Universal Master Mix kit (Applied Biosystems, Foster City, CA, United States), according to the instructions of the manufacturer, with 2.5 μL of sample DNA. The DNA was amplified using the 7500 Real Time PCR system (Applied Biosystem) followed by 40 cycles of 95°C for 15 s and 60°C for 1 min. Positive samples were defined as those with cycle threshold (*CT*) values <40.

The conventional PCR reaction volume of 25 μL contained the following components: 12.5 μL of 2 × Taq PCR MasterMix (Tiangen Biotech Co. Ltd., Beijing, China), 0.4 μM each primer, and 2 μL extracted DNA. The reaction consisted of one cycle at 95°C for 10 min, followed by 35 cycles at 95°C for 30 s, 55°C for 30 s, and 72°C for 1 min, with a final extension at 72°C for 10 min. The primer set *lytA*-F (5′-GCTAATGCCCCATTTAGCAA-3′) and *lytA*-R (5′-CTATGCAGCGGTTGAACTGA-3′) defined an amplicon of 235 bp. The PCR products were visualized on a 1.5% agarose gel and stained with GeneGreen. Images were obtained on the Gel Doc EQ imaging system (Bio-Rad). The amplified products were sequenced by Sangon Biotech (Shanghai) Co., Ltd. The sequences were analyzed using BLAST.

### Clinical Specimen Collection and Testing

The LAMP assays and PCR assays were performed simultaneously to detect *S. pneumoniae* in the clinical samples. All assays with each clinical sample were performed in triplicate. *M. pneumoniae* in the *S. pneumoniae*-positive specimens were detected using the *Mycoplasma pneumoniae* Diagnostic Kit (Isothermal RNA amplification, Shanghai Rendu Biotechnology Co., Ltd.). All nasopharyngeal samples were plated on blood agar and chocolate agar plates and incubated at 37°C in an atmosphere of 5% CO_2_ for 24 h. *S. pneumoniae* was confirmed by mass spectrometry identification test and PCR for *lytA* gene as described earlier. *S. pneumoniae* isolates were screened according to the protocols described on the MLST website.^3^ The following housekeeping genes were detected: *aroE*, *gdh*, *gki*, *recP*, *spi*, *xpt*, and *ddl* (Enright and Spratt, 1998). The allelic number (sequence type, ST) was determined. The clonal complexes, were determined using eBURST.^4^ Besides *lytA* and *psaA*, seven major virulence genes-*cbpA*, *cps2A*, *nanA*, *pavA*, *piaA*, *ply*, and *spxB*, were also detected *via* PCR with oligonucleotide primers. The reaction system and conditions were the same as those described above.

## Results

### Optimizing the Loop-Mediated Isothermal Amplification Assay

**FIGURE 1:**
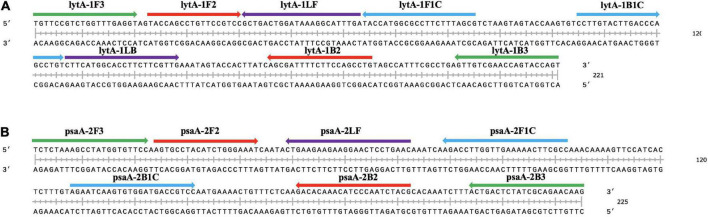
Selection of specific regions and primer positions. **(A)** Primers to amplify the *lytA* gene. **(B)** Primers to amplify the *psaA* gene. F3: outer forward primer; B3: outer backward primer; FIP: forward inner primer, F1c-F2; BIP: backward inner primer, B1c-B2; LF: loop forward primer; LB: loop backward primer.

**FIGURE 2:**
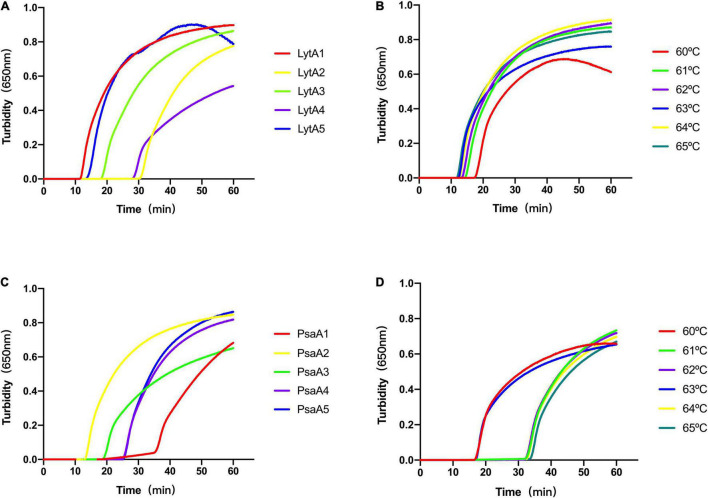
The most suitable primers and reaction temperature used for loop-mediated isothermal amplification (LAMP) analysis. **(A)** The most suitable primer pair for LAMP analysis to amplify the *lytA* gene. **(B)** The optimal reaction temperature for LAMP analysis using primer LytA-1. **(C)** The most suitable primer set for LAMP analysis to amplify the *psaA* gene. **(D)** The optimal reaction temperature for LAMP determination using primer PsaA-2. The reaction volume is 25 μL, contains 2 μL of DNA template, and the template concentration was 2 ng/μL.

Recombinant LytA and PsaA plasmids were used as positive templates, and deionized distilled water as a negative template to optimize LAMP reaction conditions. All reactions were performed in triplicate. Primer sets LytA-1, LytA-2, LytA-3, LytA-4, and LytA-5 for *lytA* gene and PsaA-1, PsaA-2, PsaA-3, PsaA-4, and PsaA-5 for *psaA* gene were compared, and turbidity was monitored for 60 min and the results were displayed (Figure 1 and Supplementary Table 1). Under the same temperature (63°C) and reaction system, range of the five primer sets were LytA-1, LytA-2, LytA-3, LytA-4, and LytA-5; PsaA-2, PsaA-1, PsaA-3, PsaA-4, and PsaA-5, according to the speed and amplification efficiency. The primer sets LytA-1 and PsaA-2 amplified the gene in the shortest time at the high template concentration, with an average time of 13 and 15 min, respectively (Figure 2). Therefore, these two sets of primers were selected as the best primers for LAMP detection.

The optimal reaction temperature range of *Bst* enzyme is 60–65°C. We used LytA-1 and PsaA-2 as primers, changed the reaction temperature to compare the amplification efficiency of LAMP, and determined the best reaction temperature in a 25-μL system (Figure 2). At 63°C, the best amplification efficiency was observed for two target genes with an average time of 13 and 15 min, respectively. Thus, 63°C was considered to be the optimal temperature for the novel LAMP reaction to simultaneously amplify two target genes.

### Sensitivity Test for the Loop-Mediated Isothermal Amplification Assay

**FIGURE 3:**
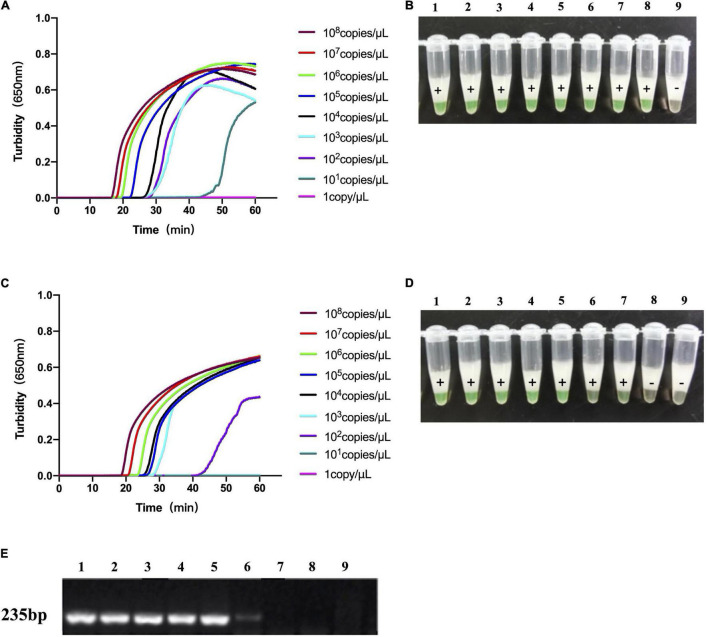
The sensitivity of loop-mediated isothermal amplification (LAMP) analysis and conventional PCR to the detection of *S. pneumoniae*. **(A,B)** The sensitivity of LAMP analysis using the primer set LytA-1 to target the *lytA* gene. **(C,D)** Sensitivity of LAMP assay using primer set PsaA-2 to target *psaA* gene. **(E)** The sensitivity of conventional PCR detection for *lytA* and *psaA* gene. Use Loopamp real-time turbidity meter to monitor and detect turbidity **(A,C)**, and judge with the naked eye based on the color change from orange to green **(B,D)**. Lanes 1–9: 10^8^, 10^7^, 10^6^, 10^5^, 10^4^, 10^3^, 10^2^, 10^1^, and 10^0^copies/μL.

The template concentration of the recombinant LytA and PsaA plasmids measured by NanoDrop spectrophotometer before use was 148.9 and 169.3 ng/μL, respectively. A 10-fold dilution with deionized water was carried out, with the concentration ranging from 10^8^ to 1 copy/μL. We used 10-fold dilutions of LytA and PsaA plasmids for the LAMP reaction. The test results were shown in Figure 3. Both the turbidimeter test and the naked-eye color method test had similar results. LytA-1 primer set took 18 min to detect 10^8^copies/μL and 50 min to detect 10 copies/μL. For the primer set PsaA-2, it took 20 min to detect 10^8^copies/μL and 50 min to detect 100 copies/μL. Therefore, using the primer sets LytA-1 and PsaA-2 within 60 min at 63°C, the detection sensitivity of each reaction was 10 and 100 copies/μL, respectively. The detection limit was the same when observed visually; all positive reactions turned green, while the negative reactions remained orange. The lowest concentration that could be detected by conventional PCR was 10^3^copies/μL using the primers targeting *lytA*. The LAMP method was 100-fold more sensitive than conventional PCR.

### Specificity Test for Loop-Mediated Isothermal Amplification Assay

**FIGURE 4:**
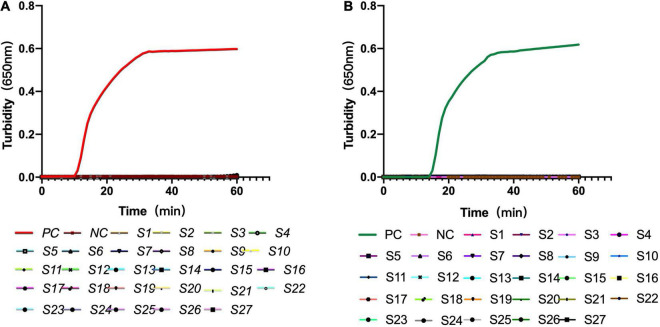
Specificity of the loop-mediated isothermal amplification assay for *S. pneumoniae* detection. **(A)** Specificity of the LAMP assay with primer set LytA-1 targeted the *lytA* gene. **(B)** Specificity of the LAMP assay with primer set PsaA-2 targeted the *psaA* gene. The detection was monitored by turbidity using a Loopamp real-time turbidimeter. S1: influenza A, S2: influenza B, S3: parainfluenza viruses, S4: adenoviruses, S5: respiratory syncytial virus, S6: human metapneumovirus, S7: human bocavirus, S8: rhinovirus, S9: *Mycoplasma pneumoniae* M129, S10: *Mycoplasma pneumoniae* FH, S11: *Haemophilus influenzae*, S12: *Legionella pneumophila*, S13: *Listeria monocytogenes*, S14: *Staphylococcus aureus*, S15: *Klebsiella pneumoniae*, S16: *Pseudomonas aeruginosa*, S17: *Escherichia coli*, S18: *Acinetobacter baumannii*, S19: *Mycobacterium tuberculosis*, S20: *Bordetella pertussis*, S21: *Streptococcus mitis*, S22: *Streptococcus oralis*, S23: *Streptococcus agalactiae*, S24: *Streptococcus mutans*, S25: *Streptococcus parasanguinis*, S26:*Streptococcus sanguinis*, S27:*Streptococcus salivarius.*

The constructed recombinant LytA and PsaA plasmids were used as templates, and deionized distilled water was used as a negative control to test the specificity of LytA-1 and PsaA-2. Both real-time turbidity meter and naked-eye coloring results found that the primer only caused amplification of *S. pneumoniae* but not the other 27 strains and deionized distilled water (Figure 4). Therefore, the LAMP assay showed no cross-reactivity with other common pathogens.

### Clinical Sample Detection

LAMP, conventional PCR, and real-time PCR were simultaneously used to detect *S. pneumoniae* in 748 samples obtained from individuals with pneumonia. The detection results of the conventional PCR were consistent with real-time PCR. Among them, 135 (18.0%) specimens were confirmed to be positive for *S. pneumoniae* and 613 were negative by the LAMP assay, 129 (17.2%) specimens were confirmed to be positive and 619 were negative by the other two PCR assays. Thus, 99.2% detection results of the three methods were consistent (Table 2). The sensitivity was 100% (95% CI 96.4–100%) and specificity was 99.0% (95% CI 97.8–99.6%). Six samples detected positive by LAMP assay were negative by conventional PCR assays. Of the 135 *S. pneumoniae*-positive clinical samples, 37.0% (50/135) were also positive for *M. pneumoniae*.

**TABLE 2:** Comparison between loop-mediated isothermal amplification assay and polymerase chain reaction for *Streptococcus pneumoniae* detection from clinical samples.

Results	LAMP		Traditional PCR/real-time PCR
	*lytA* gene	*psaA* gene	
Positive samples	135 (Time: 20–45 min)	135 (Time: 28–50 min)	129 (*Ct* < 40)
Negative samples	613 (no change in color)	613 (no change in color)	619 (no peak)

### Molecular Characteristics of the *S. pneumoniae* Isolates

The molecular characteristics of *S. pneumoniae* isolates within individual patients were shown in Table 3. We collected 22 *S. pneumoniae* isolates from the positive specimens and divided them by MLST analysis into 14 distinct STs; one of which was newly assigned. The new ST type was a novel combination of known alleles, *aroE15*, *gdh29*, *gki4*, *recp21*, *spi30*, *xpt1*, and *ddl300*. The predominant STs were ST 320 (22.7%, 5/22), ST271 (13.6%, 3/22), ST90 (9.09%, 2/22), and ST876 (9.09%, 2/22). ST320 and ST271 belong to the same clonal complexes-CC271 (36.4%, 8/22). The remaining 10 isolates belonged to 10 distinct types. All isolates carried virulence genes-*lytA*, *psaA*, *pavA*, *ply*, *spxB*, and *piaA*. Nineteen (86.4%) isolates carried *nanA*, 18 (81.8%) isolates carried *cps2A*, 16 (72.7%) isolates carried *cbpA*.

**TABLE 3:** Characteristics of *Streptococcus pneumoniae* isolates.

Isolate	Source	MLST type	*lytA*	*psaA*	*cbpA*	*nanA*	*cps2A*	*pavA*	*ply*	*spxB*	*piaA*
Isolate 1	Swab	ST90	+	+	+	+	+	+	+	+	+
Isolate 2	Swab	ST271	+	+	+	–	–	+	+	+	–
Isolate 3	Swab	ST143	+	+	+	+	+	+	+	+	+
Isolate 4	Sputum	ST386	+	+	+	+	+	+	+	+	+
Isolate 5	Sputum	ST280	+	+	+	–	+	+	+	+	+
Isolate 6	Sputum	NEW	+	+	–	+	–	+	+	+	+
Isolate 7	Sputum	ST1416	+	+	+	+	+	+	+	+	+
Isolate 8	Sputum	ST90	+	+	–	+	+	+	+	+	+
Isolate 9	Sputum	ST320	+	+	+	+	+	+	+	+	+
Isolate 10	Sputum	ST876	+	+	+	+	+	+	+	+	+
Isolate 11	Sputum	ST16327	+	+	+	+	+	+	+	+	+
Isolate 12	BALF	ST1512	+	+	+	+	+	+	+	+	+
Isolate 13	BALF	ST320	+	+	+	+	+	+	+	+	+
Isolate 14	BALF	ST13529	+	+	+	+	–	+	+	+	+
Isolate 15	BALF	ST320	+	+	–	+	+	+	+	+	+
Isolate 16	BALF	ST320	+	+	+	+	+	+	+	+	+
Isolate 17	BALF	ST81	+	+	+	+	+	+	+	+	+
Isolate 18	BALF	ST3397	+	+	–	+	–	+	+	+	+
Isolate 19	BALF	ST271	+	+	+	–	+	+	+	+	+
Isolate 20	BALF	ST320	+	+	–	+	+	+	+	+	+
Isolate 21	BALF	ST 876	+	+	–	+	+	+	+	+	+
Isolate 22	BALF	ST 271	+	+	+	+	+	+	+	+	+

*lytA, autolysin; psaA, pneumococcal surface antigen A; cbpA, choline-binding protein A; nanA, neuraminidase A; cpsA, capsular polysaccharide expression regulator A; pavA, pneumococcal adhesion and virulence A; ply, pneumolysin; spxB, pyruvate oxidase; piaA, pneumococcal iron acquisition A; BALF, bronchoalveolar lavage fluid.*

## Discussion

The burden of disease caused by *S. pneumoniae* worldwide is serious, and the distribution of *S. pneumoniae* pathogenic serotypes varies from region to region. *S. pneumoniae* has >99% 16S rRNA gene homology with *Streptococcus pseudopneumoniae*, *S. mitis*, and *S. oralis* (Scholz et al., 2012). Therefore, the rapid, simple, accurate detection of *S. pneumoniae* plays a crucial role in early etiological diagnosis and effective targeted therapy. At present, it is difficult to isolate and identify *S. pneumoniae* using classical techniques, such as culture, colonial morphology, serotype, optochin susceptibility, and bile solubility. Currently, molecular diagnosis is preferred as an alternate gold standard. The PCR-based assays for detecting *S. pneumoniae* use primers specific to repetitive regions, rRNA, or genes encoding virulence, including pneumolysin, autolysin, Spn9802, capsular polysaccharide biosynthesis, and pneumococcal surface antigen A (Tettelin et al., 2001). However, the requirement for expensive equipment and trained personnel mean that these methods remain in advanced laboratories. Since its development in 2000, LAMP technology has made many achievements in diagnosis and detection, mainly including the diagnosis of clinical diseases, the qualitative and quantitative detection of epidemic bacteria or viruses, and the sex identification of animal embryos. LAMP is a powerful tool for rapid diagnosis of infectious diseases.

Autolysin (N-acetylmuramoyl-L-alanine amidase) gene *lytA* is one of the cell wall hydrolytic enzymes, and acts as a virulence factor through release of highly inflammatory cell wall degradation products and pneumolysin from the cytoplasm. PsaA is part of an ABC transporter and a substrate-binding lipoprotein. PsaA is also a key factor for adhesion to cells, virulence, and response to oxidative stress. In this study, to improve the sensitivity, we selected two target genes *lytA* and *psaA* to establish the LAMP method. When designing the primer sets, we analyzed and compared the *lytA* and *psaA* gene sequences in 13 *S. pneumoniae* serotypes involved in the 13-valent pneumococcal conjugate vaccine (Supplementary Figure 1). In order to ensure the stability of the test, we selected the *lytA* gene (221 bp) and *psaA* gene (225 bp) conversion regions without genetic mutations to design the primers. Although there are multiple *lytA* fragments in some serotype strains, at least one type of *lytA* gene can be detected with our new LAMP method. To accelerate the reaction, we also designed loop primers LF and LB. After isothermal incubation with strand displacement polymerase, five/six primers for seven/eight regions produced self-priming dumbbell-shaped templates, which quickly produced a large number of complex amplicons.

The isothermal amplification established in this study does not involve the process of changing temperature, and only a constant temperature device can achieve the detection purpose (Wong et al., 2018). In this method, the *lytA* and *psaA* fragments were detected when they were amplified using 10 and 100 copies/μL templates. The sensitivity was consistent with previous studies that used other assays (Dao Thi et al., 2020). A dual system with two target genes has superior sensitivity to traditional PCR and RT-PCR that use one target gene (Huang et al., 2020). LAMP has lower cost (2 United States dollar/sample, prepare the premix by ourselves) and faster detection speed (<1 h) compared with traditional detection methods (15 United States dollars/sample and >2 h detection speed). The novel LAMP assay presented positive results in around 10 min when biological samples at high concentration were used. The detection limit for turbidity was the same as that for visual observation. Owing to visualization, the detection result of LAMP method is more convenient to judge by color change from green to orange. Our novel method had no cross-reaction with 27 other pathogens, and the specificity was better than that reported for the serology test (Eldin et al., 2019). We used seven different *Streptococcus* species to verify the specificity of our method, which showed that it had superior specificity to distinguish *S. pneumoniae* from same species. To assess the applicability of the assay for the clinical diagnosis of *S. pneumoniae*, we evaluated 748 clinical samples. The sensitivity of our LAMP assay was 100% (95% CI 96.4–100%) and its specificity was 99.0% (95% CI 97.8–99.6%), which showed that it was superior to many other reported detection methods. For the six samples detected negative by conventional and real-time PCR, it took 40–50 min by LAMP assay using the Real-time Turbidimeter, and the peak value was relatively low. All these indicate that the numbers of *S. pneumoniae* in these six samples was very low, so conventional and real-time PCR did not detect to be positive.

The MLST approach has been used to monitor the molecular epidemiology of bacteria since 1998 (Maiden et al., 1998). In the present study, the prevalent STs were ST320, ST271, ST876, and ST90. ST320 was found to be the predominant type in serotype 19A isolated from 10 Asian countries (Fu et al., 2019). According to eBURST analysis, both ST320 and ST271 belonged to clonal complex 271 (36.4%, 8/22), which emerged in the United States after the widespread use of conjugate vaccine PCV7 (Ko and Song, 2004).

Among the *S. pneumoniae*-positive samples, 37.0% were co-infected with *M. pneumoniae. M. pneumoniae* is one of the most important pathogens in community-acquired pneumonia in children, and can cause severe disease. Co-infection with *S. pneumoniae* and *M. pneumoniae* can aggravate pneumonia and even lead to death in pediatric patients.

The strength of the current study was that the LAMP assay yielded accurate results within 1 h. The newly developed LAMP assay could be performed without skilled personnel or specialized instruments, and positive results could be judged by the naked eye according to a color change from orange to green, illustrating the obvious benefits of this assay. However, a limitation of the study was the lack of isolates to analyze the characteristics of *S. pneumoniae* in children, and this needs to be verified by collection of more clinical samples from children. The probability and propensity of co-infection with *M. pneumoniae* also need to be further explored.

In conclusion, our LAMP-based method showed a good diagnostic performance. The method is simple, rapid, visual, and highly sensitive and specific for the diagnosis of *S. pneumoniae* infection in clinical samples. LAMP detection is a powerful tool for *S. pneumoniae* identification, not only in diagnostic laboratories, but also in resource-constrained settings, because it does not require complex equipment.

## Data Availability Statement

The original contributions presented in the study are included in the article/Supplementary Material, further inquiries can be directed to the corresponding authors.

## Author Contributions

JJY and CY designed the study and revised the manuscript. SD, CY, BD, HF, QunZ, NL, RZ, SL, XL, XC, and YZ performed the experiments. GX, PZ, HZ, and YF collected the clinical samples. SD, CY, JC, LG, JF, ZF, TF, ZX, and QiZ analyzed the results. SD and CY wrote the manuscript. All authors read and approved the final manuscript.

## Conflict of Interest

The authors declare that the research was conducted in the absence of any commercial or financial relationships that could be construed as a potential conflict of interest.

## Publisher’s Note

All claims expressed in this article are solely those of the authors and do not necessarily represent those of their affiliated organizations, or those of the publisher, the editors and the reviewers. Any product that may be evaluated in this article, or claim that may be made by its manufacturer, is not guaranteed or endorsed by the publisher.

## Footnotes

1
https://www.ncbi.nlm.nih.gov/

2
http://primerexplorer.jp/e/

3
http://pubmlst.org/spneumoniae/

4
http://eburst.mlst.net
