# Supplementary material for: Development of Loop-Mediated Isothermal Amplification Assay Targeting lytA and psaA Genes for Rapid and Visual Diagnosis of Streptococcus pneumoniae Pneumonia in Children
Source: Front Microbiol. 2022 Jan 17;12:816997. doi: 10.3389/fmicb.2021.816997 (PMC8803124; doi:10.3389/fmicb.2021.816997)

Supplementary FIGURE 1

Sequence comparison and gene analysis of the *lytA* and *psaA* genes in different *Streptococcus pneumoniae serotypes*

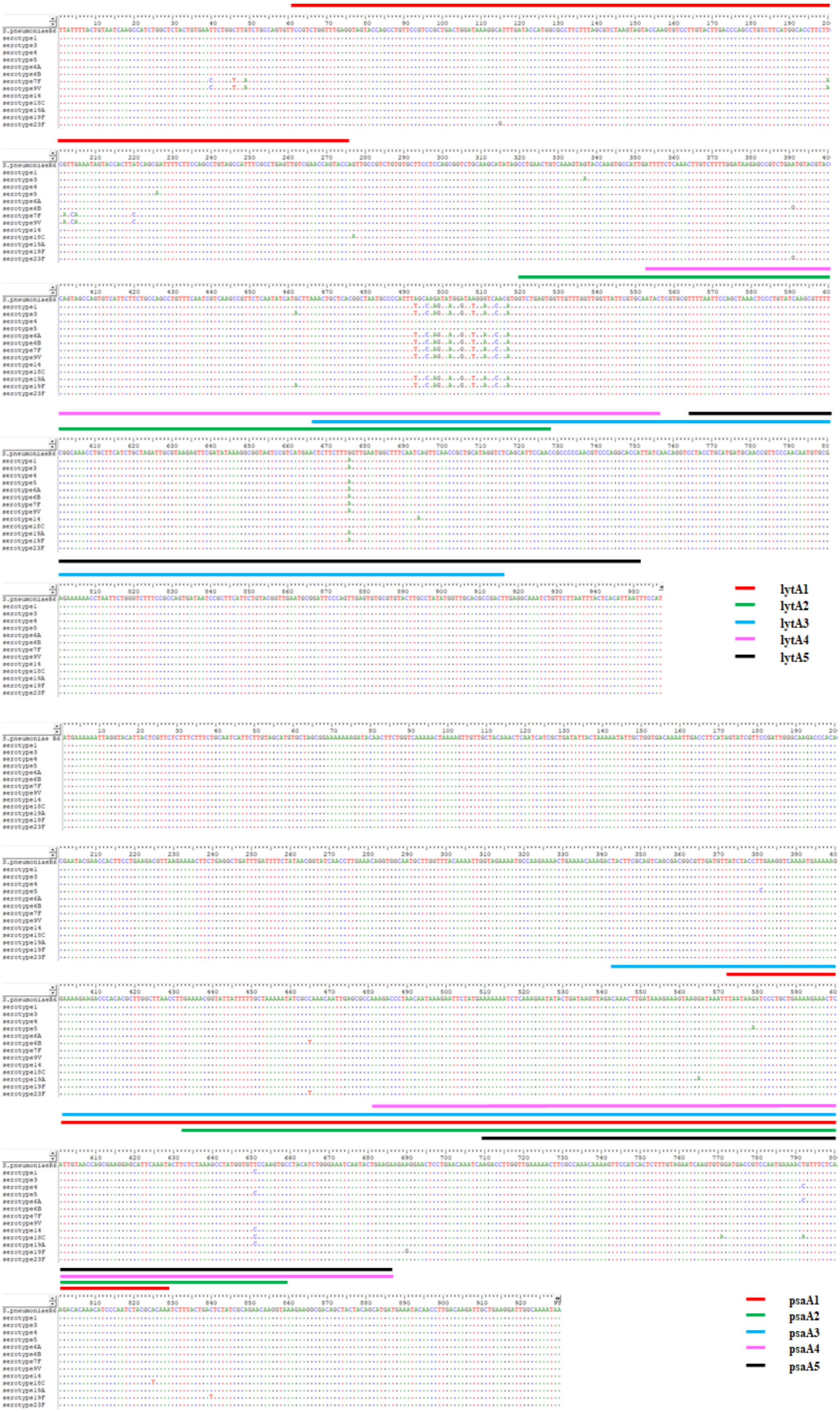

Supplement: Supplementary file 1 [file Image_1.pdf]
