# Supplementary material for: Development of Loop-Mediated Isothermal Amplification Assay Targeting lytA and psaA Genes for Rapid and Visual Diagnosis of Streptococcus pneumoniae Pneumonia in Children
Source: Front Microbiol. 2022 Jan 17;12:816997. doi: 10.3389/fmicb.2021.816997 (PMC8803124; doi:10.3389/fmicb.2021.816997)
Supplement: Supplementary file 2 [file Table_1.pdf]

Supplementary TABLE 1
Primers used for the loop-mediated isothermal amplification (LAMP) assay to detect *S.pneumoniae* detection

Primer	Sequence （5'-3'）
LytA gene	
LytA-1F3	TGTTCCGTCTGGTTTGAGGT
LytA-1B3	ACTGGTACTGGTTCGACAAC
LytA-1FIP	CGCTAAAGAAGGCGCCATGGTATACCAGCCTGTTCCGTCC
LytA-1BIP	CCTTGTACTTGACCCAGCCTGTCAGGCTGGAAGAAAATCGCT
LytA-1LF	TCAAATGCCTTTATCCAGTCAGC
LytA-1LB	CTTCATGGCACCTTCTTCGTTG
LytA-2F3	GGTCTGAGTGGTTGTTTGGT
LytA-2B3	GCGGTTGGAATGCTGAGAC
LytA-2FIP	AGCAGGTTTGCCGAAAACGCTATTCGTGCAATACTCGTGCG
LytA-2BIP	AGGCGGTAGTCCGTCATGAACTTGCAGCGGTTGAACTGATTG
LytA-3F3	GCTGCATAGGTCTCAGCATT
LytA-3B3	GCGTGCAACCATATAGGCAA
LytA-3FIP	TCGCACATTGTTGGGAACGGTTTCCCAGGCACCATTATCAAC
LytA-3BIP	TCTGGGTCTTTCCGCCAGTGACTCAACTGGGAATCCGCATT
LytA-4F3	GGTCTGAGTGGTTGTTTGGT
LytA-4B3	GTTGATAATGGTGCCTGGGA
LytA-4FIP	AGCAGGTTTGCCGAAAACGCTTCGTGCAATACTCGTGCG
LytA-4BIP	AGGCGGTAGTCCGTCATGAACTTGGAATGCTGAGACCTATGC

LytA-4LB	TGGCTTTCAATCAGTTCAACCGC
LytA-5F3	GCATAGGTCTCAGCATTCCA
LytA-5B3	AGTCGGCGTGCAACCATA
LytA-5FIP	CTCGCACATTGTTGGGAACGGTTCCCAGGCACCATTATCAAC
LytA-5BIP	TCTGGGTCTTTCCGCCAGTGAGCAAGTACACGCACACTCAA
LytA-5LF	TGCATCATGCAGGTAGGACC
LytA-5LB	ATCCGCTTCATTCTGTACGGTTGAA
***PsaA* gene**	
PsaA-1F3	GATCCCTGCTGAAAAGAAACTC
PsaA-1B3	CGTAGATTGGGATGTTTGTGTC
PsaA-1FIP	TCCCAGATGTAGGCACTTGGAACACCAGCGAAGGAGCATTCA
PsaA-1BIP	AGACCTTGGTTGAAAAACTTCGCCGGACGGTCATCCACACTT
PsaA-2F3	TCTCTAAAGCCTATGGTGTTCC
PsaA-2B3	CTTGTTCTGCGATAGAGTCAGT
PsaA-2FIP	GGCGAAGTTTTTCAACCAAGGTCTAGTGCCTACATCTGGGAAAT
PsaA-2BIP	AGAATCAAGTGTGGATGACCGTCCCGTAGATTGGGATGTTTGTGTC
PsaA-2LF	GTTCAGGAGTTCCTTCTTCTTCAG
PsaA-3F3	GAAACTCATTGTAACCAGCG
PsaA-3B3	TGTGCGTAGATTGGGATG
PsaA-3FIP	GTTCAGGAGTTCCTTCTTCTTCAGCTCTAAAGCCTATGGTGTTCC
PsaA-3BIP	CCTTGGTTGAAAAACTTCGCCATTTCATTGGACGGTCATCC
PsaA-3LF	TGATTTCCCAGATGTAGGCACT

PsaA-4F3	CTGAAGAAGAAGGAACTCCTGAAC
PsaA-4B3	TCATGCTGTAGTAGCTGTCG
PsaA-4FIP	GGACGGTCATCCACACTTGATTCTACCTTGGTTGAAAAACTTCGCC
PsaA-4BIP	AGACACAAACATCCCAATCTACGCCCTTCTTTACCTTGTTCTGCG
PsaA-5F3	CCTGAACAAATCAAGACCTTGG
PsaA-5B3	TCATGCTGTAGTAGCTGTCG
PsaA-5FIP	ACAGTTTTCATTGGACGGTCATCCCGCCAAACAAAAGTTCCATCAC
PsaA-5BIP	AGACACAAACATCCCAATCTACGCCCTTCTTTACCTTGTTCTGCG

F3: outer forward primer; B3: outer backward primer; FIP: forward inner primer; BIP: backward inner primer; LF: loop forward primer; LB: loop backward primer; FIP:F1c-F2; BIP: B1c-B2.
