# Supplementary material for: Development of Loop-Mediated Isothermal Amplification Assay Targeting lytA and psaA Genes for Rapid and Visual Diagnosis of Streptococcus pneumoniae Pneumonia in Children
Source: Front Microbiol. 2022 Jan 17;12:816997. doi: 10.3389/fmicb.2021.816997 (PMC8803124; doi:10.3389/fmicb.2021.816997)
Supplement: Supplementary file 3 [file Table_2.pdf]

Supplementary TABLE 2

Primers used for detection of *S.pneumoniae* virulence genes

Virulence gene	Primer sequence (5'-3')	product length (bp)
cbpA[1]	F: CGAGGGCACAATTAAGCAAGCA<br>R: CGATCTGTCTTGATGTTTTCTAACCT	570
nanA[1,2]	F: AGAGGGGCGTGGTTCGGATGGGAAACAG<br>R: ACTCGGTCACCCCAAGTTTTACCATTA	600
cps2A[1]	F: GTAGAAGCTCCTAAGACGTCTA<br>R: GAAGGATAGCCACAATCACCT	517
pavA[3]	F: TGCAAATCAGGCGGGAGAGCCTT<br>R: TGGATTTTCTCCCGTTGTCTTCT	540
ply[1]	F: ACGGCTCACAGCATGGAACAACTC<br>R: TACAACTCGGGCACCCGAACTTGG	450
spxB[1]	F: ACCAGCCAACGAAGTGGTCTTTGAA<br>R: TGGCGATCTGGATTGTCTTTCTTA	516
piaA[4]	F: CTTCTAAAGAGCATGCGCTTG<br>R: CTGGAATTACAGCAACGGC	828

[1] LeMessurier, K.S., Ogunniyi, A.D., and Paton, J.C. (2006). Differential expression of key pneumococcal virulence genes in vivo. Microbiology (Reading) 152(Pt 2), 305-311. doi: 10.1099/mic.0.28438-0

[2] Ding, F., Tang, P., Hsu, M.H., Cui, P., Hu, S., Yu, J., et al. (2009). Genome evolution driven by host adaptations results in a more virulent and antimicrobial-resistant Streptococcus pneumoniae serotype 14. BMC Genomics 10, 158. doi: 10.1186/1471-2164-10-158.

[3] Lin, F.P., Lan, R., Sintchenko, V., Gilbert, G.L., Kong, F., and Coiera, E. (2011). Computational bacterial genome-wide analysis of phylogenetic profiles reveals potential virulence genes of Streptococcus agalactiae. PLoS One 6(4), e17964. doi: 10.1371/journal.pone.0017964.

[4] Mitchell, A.M., and Mitchell, T.J. (2010). Streptococcus pneumoniae: virulence factors and variation. Clin Microbiol Infect 16(5), 411-418. doi: 10.1111/j.1469-0691.2010.03183.x
